# Work-related stress and well-being in association with epigenetic age acceleration: A Northern Finland Birth Cohort 1966 Study

**DOI:** 10.18632/aging.203872

**Published:** 2022-02-02

**Authors:** Anna Freni-Sterrantino, Giovanni Fiorito, Angelo D’Errico, Oliver Robinson, Marianna Virtanen, Leena Ala-Mursula, Marjo-Riitta Järvelin, Justiina Ronkainen, Paolo Vineis

**Affiliations:** 1 MRC Centre for Environment and Health, Department of Epidemiology and Biostatistics, Imperial College London, St Mary's Campus, London W2 1PG, United Kingdom; 2 Laboratory of Biostatistics, Department of Biomedical Sciences, University of Sassari, Sassari 07100, Italy; 3 Department of Epidemiology, Local Health Unit TO 3, Turin 10095, Italy; 4 School of Educational Sciences and Psychology, University of Eastern Finland, Joensuu FI-80101, Finland; 5 Division of Insurance Medicine, Karolinska Institutet, Stockholm 17177, Sweden; 6 Center for Life Course Health Research, Faculty of Medicine, University of Oulu, Oulu 90014, Finland; 7 Grantham Institute for Climate Change and School of Public Health, Imperial College London, London SW7 2AZ, United Kingdom; 8 IIGM – Italian Institute for Genomic Medicine (IIGM), IRCCS Candiolo, Torino 10060, Italy

**Keywords:** epigenetic age, job strain, effort-reward imbalance, work-related well-being, DNA methylation

## Abstract

Recent evidence indicates consistent association of low socioeconomic status with epigenetic age acceleration, measured from DNA methylation. As work characteristics and job stressors are crucial components of socioeconomic status, we investigated their association with various measures of epigenetic age acceleration.

The study population included employed and unemployed men and women (n=604) from the Northern Finland Birth Cohort 1966. We investigated the association of job strain, effort-reward imbalance and work characteristics with five biomarkers of epigenetic aging (Hannum, Horvath, PhenoAge, GrimAge, and DunedinPoAm).

Our results indicate few significant associations between work stress indicators and epigenetic age acceleration, limited to a range of ±2 years, and smoking recording the highest effect on GrimAge age acceleration biomarker between current and no smokers (median difference 4.73 years (IQR 1.18, 8.41). PhenoAgeAA was associated with job strain active work (β=-1.301 95%CI -2.391, -0.212), slowing aging of less than 1.5 years, and working as white-collar slowed aging six months (GrimAgeAA β=-0.683, 95%CI -1.264, -0.102) when compared to blue collars. Association was found for working for more than 40 hours per week that increased the aging over 1.5 years, (HorvathAA β =2.058 95%CI 0.517,3.599, HannumAA β=1.567, 95%CI 0.415,2.719).

The pattern of associations was different between women and men and some of the estimated effects are inconsistent with current literature. Our results provide the first evidence of association of work conditions with epigenetic aging biomarkers. However, further epidemiological research is needed to fully understand how work-related stress affects epigenetic age acceleration in men and women in different societies.

## INTRODUCTION

DNA methylation-based biomarkers of biological age [1] - epigenetic clocks - have become very popular and have been found to be associated with several risk factors for non-communicable diseases and longevity [2]. Epigenetic clocks are a composite score of DNA methylation levels at different CpG sites in the genome. The differences between epigenetic clocks and chronological age have been defined as epigenetic age acceleration (EAA). Positive values of EAA indicate that an individual is experiencing accelerated aging. EAA has been found to predict all-cause mortality, frailty, psychosocial stress [3, 4], cardiovascular disease [5], diabetes, cancer [1, 6], and higher values are associated with a decline in cognitive ability, depression and anxiety [7, 8].

Previous studies [9–13] has suggested that low socio-economic status (SES) is associated with EAA, using different proxies such as education, parental occupation, income, and combined measures like the relative index of inequality. It emerges that the detrimental effect of low SES positions starting early in life are detectable through epigenetic clocks [14]. Based on recent literature, EAA has been suggested as an intermediate biological mechanism linking environmental exposures (including stress) with poor health outcomes and mortality later in life.

However, most studies investigated older populations, while only a few studies have investigated younger subjects. Based on a sample of middle-aged women, Simons et al. [15] have found an association between income and accelerated aging, that was unaffected by controlling for other SES-related factors (i.e., education, marital status, and childhood adversity). They concluded that chronic financial pressures associated with low income exert a weathering effect that results in premature aging. Using data on age acceleration from the UK Household Longitudinal Study, Hughes et al. [16] confirmed the association of EEA and low SES in early life, but no associations were found with current/adult measures of social position on a sample aged less than 65 years old.

Little is known whether current occupational characteristics or job-related stress - crucial SES characteristics - are associated with EAA. Among the few available studies, a recent study reported an increase in the GrimAge marker, but not in the other two AA markers (DNAm age, PhenoAge), associated with high occupational physical activity [17]. In a US study, performing shift work or night shift work for more than 10 years was associated with an increased EAA PhenoAge marker [10]. Carugno et al. [18], in a study on female nurses, found that night shift work was associated with an increased age acceleration, measured through analysis of five CpG islands in five genes, but limited to subjects with overweight/obesity or exposed to high work stress.

DNA methylation levels (not EAA) have been associated with stress-related conditions. Based on their systematic review of human and animal studies on work stress, burnout and depression, Bakusic et al. [19] concluded that DNA methylation changes are possible biomarkers of stress-related mental disorders. Among human studies, Duman et al. [20] reported a significant increase in global DNA methylation associated with perceived work stress. In a Finnish study on nurses, hypomethylation of the promoter of the serotonin transporter gene (SLC6A4) was associated with both increased burnout symptoms and perceived work stress when mutually adjusted for [21]. Furthermore, in a Japanese study on manufacturing workers, methylation of most CpG islands in the tyrosine hydroxylase gene, promoter included, was significantly increased among those exposed to high job strain [22].

There is ample evidence from the literature on work stress that exposure to psychosocial stressors in the workplace, like those measured using work-stress models such as the demand-control [23] and the effort-reward-Imbalance [24] model, is associated with clinical biomarkers and adverse outcomes. According to the former, the combination of demands and control defines the job strain; it posits that people working in jobs characterized by high demands and low control are at risk of stress-related ill health and disease. Support to this model comes from studies showing that workers exposed to high strain increased risks of coronary heart disease [23, 24], diabetes [25], atherosclerosis in its early non-symptomatic stages [26], as well as increased levels of brain-derived neurotrophic factor [27]. The effort-reward imbalance model, which stems from the social exchange theory [28], captures an employee dissatisfaction about the perceived imbalance between the reward received, in terms of money, career, recognition and job security, and the effort made that would affect well-being and health-related behaviour. In 2017, a review found that effort-reward imbalance was associated with several biological changes in pathways, leading to stress-related conditions: including decreased heart rate variability, increased blood lipids, blood pressure and cortisol release, altered immune function, inflammation, increased risk of metabolic syndrome [29]. Furthermore, a large multicohort study found an increased risk of coronary heart disease associated with high effort-reward imbalance [30].

In this context, to contribute elucidating potential mechanisms through which life-stressing conditions and job stress may impact health, we investigated the relation of EAA with psychosocial and other work characteristics. In relation to epigenetic clocks, we examined the distribution of work-related stress and well-being indicators in the Northern Finland Birth cohort 1966 (NFBC 1966), adjusting for known risk factors for accelerated ageing.

We analyzed well-known work-related stress indicators such as job strain, effort-reward imbalance and overcommitment, discontinuous work history, excessive working hours, shift work, and other work characteristics, including two indicators of positive occupational psychology: work engagement and work favouring attitude. We evaluated the epigenetic age acceleration using five epigenetic ageing biomarkers: Horvath and Hannum first-generation clocks, Levine DNA methylation PhenoAge and Lu's DNA methylation GrimAge (the last two known as the second-generation clocks), and the newly developed pace of aging biomarker, DunedinPoAm, trained on longitudinal data [1, 6, 31–33].

## RESULTS

### Descriptive summary of epigenetic age acceleration and pace of ageing

Table 1 contains detailed definitions and interpretations of the two work stress indicators and the other work characteristics examined.

**Table 1:** Job exposure definitions.

**Concept**	**Description**
**Job strain**	Depicts an individual’s experience of a psychosocial work environment that entails a high level of job demands combined with a low level of job control, resulting in job strain. Originally suggested by Karasek in 1979, the demands-control model highlights the role of job control, as not only buffering for stress but resulting in active, healthy work in circumstances where high demands and high control prevail simultaneously. A large body of evidence has linked job strain with excess morbidity and mortality [24, 34, 35]
**Effort-reward Imbalance (ERI)**	Captures an individual’s perception of reciprocity between high effort at work and the actual/expected rewards received in turn. As suggested by Siegrist in 1990s, an imbalance between these two, effort not met with sufficient rewards, is considered a source of work stress. ERI has been linked with both cardiovascular and mental health outcomes [29, 36].
**Overcommitment**	An addition to the ERI model. Represents an individual’s tendency to put high effort at work even in circumstances of low rewards. Overcommitment is considered a toxic component, increasing the work stress related to ERI.
**Occupational physical activity (OPA)**	Physical activity in the domain of work, in opposition to leisure-time physical activity. High levels of OPA (lifting, standing, heavy manual work) indicate high physical job demands. OPA may not be associated with good health, in contrast with leisure-time physical activity [37].
**Work-favouring attitude**	Refers to high personal meaning of work as a way to exercise and master skills or even as a calling, as opposed to seeing work merely as a source of income [38].
**Work engagement**	A concept of positive occupational psychology and work-related well-being [39]. Depicts experience of the following three dimensions at work: vigor, dedication and absorption (flow).
**Job security**	Captures an individual’s perception of the stability of one’s job contract [40].

Table 2 reports the descriptive statistics for the epigenetic age acceleration markers and the pace of aging at 46 years for the NFBC data. Women represent 55% of the sample, with a BMI <24.9 for 47%, mainly with a secondary education level (68%) and moderate alcohol consumption (81%), physically very active during leisure time (55%) and never smoked (53%). Men show a similar pattern; however, a higher proportion was overweight (46%). Overall, 87% had a permanent job contract. A higher proportion of men were unemployed (7.1%, compared to 4.7% among women), while women showed a higher percentage in a temporary job (10%, compared to 3% among men). We evaluated the association between job exposures and risk factors using Chi-Square test, Student t-test or ANOVA depending on the variable characteristics (categorical or continuous; see Supplementary Table 1 in Supplementary Materials). We found statistically significant association of smoking with effort and effort-reward Imbalance, type of employer (private or state/municipality) and occupational group.

**Table 2:** Descriptive statistics of the study population, mean and standard deviation (sd) for continuous variables and frequency and percentage for categorical variables.

	**All N=604**	**Females N = 337**	**Males N = 267**
HorvathAA, mean (sd)	0.0(4.0)	-0.4 (3.9)	0.5 (4.1)
HannumAA, mean (sd)	-0.01(2.99)	-0.32 (2.92)	0.39(3.04)
PhenoAgeAA, mean (sd)	-0.1(4.8)	0.2 (4.8)	-0.5 (4.8)
GrimAgeAA, mean (sd)	-0.1(4.1)	-0.8(3.9)	0.9 (4.1)
DunedinPoAm, mean (sd)	1.00(0.07)	1.01 (0.07)	1.00(0.07)
BMI n(%)			
Optimal< 24.9	248 (41%)	158 (47%)	90 (34%)
Overweight 25-29.9	232 (39%)	111 (33%)	121 (46%)
Obese >= 30	118 (20%)	64 (19%)	54 (20%)
Missing	6	4	2
Educational Level n (%)			
Basic	28 (4.7%)	15 (4.6%)	13 (4.9%)
Secondary	409 (69%)	221 (68%)	188 (71%)
Tertiary	154 (26%)	91 (28%)	63 (24%)
Missing	13	10	3
Alcohol consumption n (%)			
Never	53 (8.8%)	35 (10%)	18 (6.7%)
Moderate	493 (82%)	273 (81%)	220 (82%)
Heavy	56 (9.3%)	27 (8.1%)	29 (11%)
Missing	2	2	0
Smoking n (%)			
Never	322 (53%)	199 (59%)	123 (46%)
Past	155 (26%)	71 (21%)	84 (31%)
Current	127 (21%)	67 (20%)	60 (22%)
Physical Activity (leisure) n (%)			
Inactive	145 (24%)	71 (21%)	74 (28%)
Moderately Active	124 (21%)	66 (20%)	58 (22%)
Active/Very Active	335 (55%)	200 (59%)	135 (51%)
Job status n (%)			
Permanent	526 (87%)	286 (85%)	240 (90%)
Temporary	43 (7.1%)	35 (10%)	8 (3.0%)
Unemployed	35 (5.8%)	16 (4.7%)	19 (7.1%)
Employer			
private employer	322 (57%)	133 (42%)	189 (78%)
state/municipality	240 (43%)	186 (58%)	54 (22%)
Missing	42	18	24
Occupational group			
White collars	244 (46%)	163 (55%)	129 (54%)
Blue collars	292 (54%)	135 (45%)	109 (46%)
Missing	68	39	29
Job Control mean(sd)	3.83 (0.77)	3.77 (0.77)	3.90 (0.76)
Missing	54	31	23
Job Demands mean(sd)	4.21 (0.67)	4.31 (0.64)	4.07 (0.68)
Missing	51	27	24
Job strain Linear	0.19 (0.44)	0.27 (0.44)	0.08 (0.41)
Missing	58	33	25
Job strain n (%)			
Active work	128 (23%)	97 (32%)	77 (32%)
High strain	174 (32%)	93 (31%)	29 (12%)
Low strain	122 (22%)	53 (17%)	75 (31%)
Passive work	122 (22%)	61 (20%)	61 (25%)
Missing	58	33	25
Job strain Quotient	1.14 (0.29)	1.19 (0.30)	1.08 (0.27)
Missing	58	33	25
Job strain Tertile n (%)			
High strain	172 (32%)	131 (43%)	57 (24%)
Intermediate strain	186 (34%)	98 (32%)	88 (36%)
Low strain	188 (34%)	75 (25%)	97 (40%)
Missing	58	33	25
Effort mean(sd)	2.10 (0.67)	2.04 (0.67)	2.18 (0.66)
Missing	48	27	21
Reward mean(sd)	2.50 (0.55)	2.52 (0.54)	2.47 (0.55)
Missing	63	33	30
Effort-Reward Imbalance mean(sd)	0.90 (0.40)	0.87 (0.39)	0.94 (0.41)
Missing	66	36	30
Overcommitment mean(sd)	2.10 (0.67)	2.93 (0.65)	3.05 (0.61)
Missing	48	27	28
Employment history n (%)			
At least temporarily unemployed	269 (45%)	157 (47%)	112 (42%)
Continuously employed	333 (55%)	178 (53%)	155 (58%)
Missing	2	2	0
Occupational s Physical Activity n (%)			
Low Intensity	421 (75%)	244 (78%)	177 (72%)
Intermediate Intensity	68 (12%)	36 (12%)	32 (13%)
High Intensity	70 (13%)	33 (11%)	37 (15%)
Missing	45	24	21
Working Hours per week n (%)			
Less than 31 hours	32 (5.7%)	24 (7.7%)	8 (3.3%)
31-40 hours	402 (72%)	244 (78%)	158 (64%)
More than 40 hours	124 (22%)	45 (14%)	79 (32%)
Missing	46	24	22
Shifts n (%)			
Day job	446 (81%)	253 (82%)	193 (79%)
Evening/shift	108 (19%)	57 (18%)	51 (21%)
Missing	50	27	23
Work attitude mean(sd)	17.5 (3.5)	17.9 (3.6)	17.0 (3.3)
Missing	8	6	2
Work engagement mean(sd)	41 (10)	42 (10)	39 (11)
Missing	55	29	26
Good job security n (%)			
No	83(15%)	45(15%)	38(15%)
Yes	474 (85%)	266(85%)	208(85%)
Missing	47	26	21

**Figure 1:**
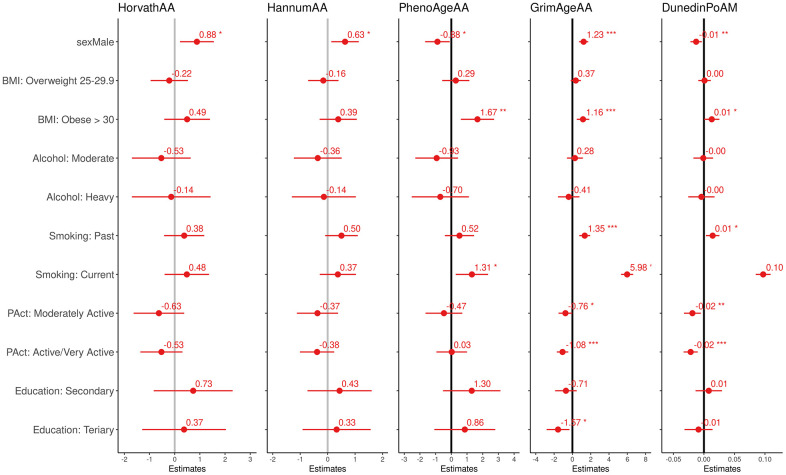
Effect size and 95% confidence intervals (interpretable as years of increase/decreasing epigenetic age and rate of aging) of the association between the four epigenetic aging biomarkers and the pace of aging and modifiable risk factor.

Additionally, we estimated the effect of non-occupational risk factors for aging on the biomarkers (sex, education, smoking habit, alcohol consumption, BMI, physical activity, see Figure 1). We observed higher EAA in men for all but PhenoAge biomarkers, which were positively associated with smoking and BMI. GrimAgeAA and DunedinPoAm also showed a positive association with smoking and BMI and a negative association with physical activity. Pearson correlations among EAA measures (Supplementary Table 2 in Supplementary Materials) show the highest coefficients were found between Horvath and Hannum AA (ρ=0.96) and between GrimAgeAA and DunedinPoAm (ρ=0.78). The remaining pairwise correlations were in the range 0.20-0.48.

### Multivariate models

Results are reported as estimates and 95% confidence intervals from linear regression, where EAA is the dependent variable and work characteristics the predictors. The estimates are interpretable as years of increase/decrease for epigenetic age: positive coefficients indicate increased age acceleration and negative coefficients indicate a decrease of the estimated biological age compared to the chronological age. For DunedinPoAm, the effect size indicates the rate of increasing biological aging (in percentage) comparing a group with the reference category. In Supplementary Materials, we have provided the results as standardized coefficients estimates for the adjusted and unadjusted models for each epigenetic biomarker (Supplementary Tables 4, 5 in Supplementary Materials); these results are interpretable with regard of the EEA standard deviations (Table 1).

### Horvath and Hannum AA

The two EEA are highly correlated, so the pattern of exposure to which they are associated are similar. In unadjusted models (Table 3), we have found that being in a temporary versus a permanent job showed a negative association with Horvath age acceleration (β = -1.28 95%CI -2.527, -0.034), hence slowing aging. Both biomarkers, Horvath and Hannum AA were associated with working for more than 40 hours per week that increased the EAA over 1.5 years, (HorvathAA β =2.058 95%CI 0.517,3.599), HannumAA β=1.567, 95%CI 0.415,2.719)) when compared with working less than 31 hours per week. In the adjusted models (Supplementary Table 3 in Supplementary Material), only good job security remained significant for HorvathAA (β=1.511 95%CI -0.001,3.022) HannumAA (β=1.171 95%CI 0.042,2.3).

**Table 3:** Unadjusted coefficient estimates form linear regression with 95% confidence intervals.

		**HorvathAA**	**HannumAA**	**PhenoAgeAA**	**GrimAgeAA**	**DunedinPoAm**
		**Estimates (95% CI)**	**Estimates (95% CI)**	**Estimates (95% CI)**	**Estimates (95% CI)**	**Estimates (95% CI)**
Job Status	permanent	Ref.	Ref.	Ref.	Ref.	Ref.
	temporary	-1.28 (-2.527,-0.034) *	-0.823 (-1.754,0.108)	0.132 (-1.365,1.629)	0.607 (-0.66,1.873)	0.014 (-0.008,0.036)
	unemployed	0.21 (-1.161,1.582)	0.105 (-0.92,1.129)	0.895 (-0.752,2.543)	1.741 (0.347,3.135) *	0.019 (-0.005,0.043)
Employer	private employer	Ref.	Ref.	Ref.	Ref.	Ref.
	state/municipality	-0.34 (-1.012,0.333)	-0.253 (-0.756,0.249)	0.187 (-0.62,0.994)	-1.219 (-1.885,-0.552) *	-0.006 (-0.017,0.006)
Occupational group	Blue collars	Ref.	Ref.	Ref.	Ref.	Ref.
	White collars	0.136 (-0.55,0.822)	0.088 (-0.425,0.602)	0.006 (-0.816,0.828)	-1.447 (-2.127,-0.768) *	-0.015 (-0.026,-0.003) *
Job control		0.328 (-0.109,0.765)	0.199 (-0.128,0.526)	-0.226 (-0.751,0.299)	-0.225 (-0.665,0.214)	-0.004 (-0.012,0.003)
Job demand		0.022 (-0.478,0.522)	0.048 (-0.325,0.422)	-0.307 (-0.907,0.292)	-0.356 (-0.857,0.145)	-0.005 (-0.013,0.004)
Job strain Linear		-0.488 (-1.257,0.281)	-0.27 (-0.845,0.305)	-0.095 (-1.014,0.823)	-0.153 (-0.921,0.615)	0 (-0.014,0.013)
Job strain	Low strain	Ref.	Ref.	Ref.	Ref.	Ref.
	active work	0.151 (-0.767,1.069)	0.169 (-0.517,0.855)	-1.201 (-2.292,-0.11) *	-0.342 (-1.258,0.573)	-0.008 (-0.024,0.008)
	passive work	0.112 (-0.886,1.109)	0.139 (-0.606,0.884)	-0.685 (-1.871,0.501)	0.21 (-0.784,1.205)	0.004 (-0.013,0.021)
	high strain	-0.355 (-1.353,0.643)	-0.225 (-0.971,0.52)	-0.262 (-1.448,0.924)	0.016 (-0.979,1.011)	0.005 (-0.012,0.022)
Job strain quotient		-0.938 (-2.087,0.211)	-0.557 (-1.417,0.302)	-0.232 (-1.606,1.143)	0.059 (-1.09,1.208)	0.001 (-0.019,0.021)
Job strain tertile	Low strain	Ref.	Ref.	Ref.	Ref.	Ref.
	Intermediate strain	-0.48 (-1.313,0.353)	-0.237 (-0.86,0.386)	-1.026 (-2.017,-0.035) *	0.041 (-0.79,0.872)	0 (-0.014,0.014)
	high strain	-0.442 (-1.273,0.389)	-0.253 (-0.875,0.368)	-0.193 (-1.182,0.795)	-0.441 (-1.27,0.388)	-0.001 (-0.015,0.014)
Effort		-0.413 (-0.909,0.084)	-0.312 (-0.683,0.059)	-0.246 (-0.848,0.356)	0.758 (0.259,1.258) *	0.009 (0.001,0.018) *
Reward		0.087 (-0.53,0.704)	0.177 (-0.284,0.639)	0.237 (-0.505,0.979)	0.327 (-0.292,0.946)	0.002 (-0.009,0.013)
Effort-Reward Imbalance		-0.524 (-1.365,0.318)	-0.451 (-1.08,0.178)	-0.396 (-1.411,0.619)	0.795 (-0.05,1.639)	0.011 (-0.003,0.026)
Overcommitment		0.171 (-0.353,0.694)	0.102 (-0.29,0.494)	0.131 (-0.498,0.76)	0.148 (-0.384,0.68)	0 (-0.009,0.009)
Work history	At least temporary unemployed	Ref.	Ref.	Ref.	Ref.	Ref.
	Continuously Employed	0.236 (-0.41,0.882)	0.238 (-0.244,0.72)	-0.11 (-0.883,0.662)	-0.665 (-1.321,-0.008) *	-0.01 (-0.021,0.001)
Occupational Physical Activity	Low Intensity	Ref.	Ref.	Ref.	Ref.	Ref.
	Intermediate Intensity	-0.282 (-1.304,0.74)	-0.149 (-0.913,0.615)	0.446 (-0.787,1.679)	1.369 (0.342,2.395) *	0.013 (-0.005,0.031)
	High Intensity	0.066 (-0.944,1.076)	0.158 (-0.597,0.913)	0.753 (-0.465,1.971)	0.68 (-0.334,1.693)	-0.003 (-0.021,0.014)
Working hours per week	less than 31 hours	Ref.	Ref.	Ref.	Ref.	Ref.
	31-40 hours	1.247 (-0.181,2.675)	0.959 (-0.108,2.026)	-0.521 (-2.254,1.213)	0.044 (-1.407,1.495)	-0.002 (-0.027,0.024)
	more than 40 hours	2.058 (0.517,3.599) *	1.567 (0.415,2.719) *	0.085 (-1.786,1.956)	0.573 (-0.994,2.139)	0.001 (-0.026,0.028)
Working shift	Day job	Ref.	Ref.	Ref.	Ref.	Ref.
	Evening/shift	0.455 (-0.383,1.293)	0.391 (-0.236,1.019)	0.585 (-0.428,1.598)	0.733 (-0.114,1.58)	0.015 (0.001,0.03) *
Work attitude		-0.004 (-0.097,0.089)	-0.01 (-0.079,0.059)	-0.006 (-0.117,0.105)	-0.041 (-0.135,0.052)	-0.001 (-0.003,0.001)
Work engagement		0.005 (-0.028,0.037)	0.003 (-0.021,0.028)	0.009 (-0.03,0.049)	-0.049 (-0.081,-0.016) *	-0.001 (-0.001,0) *
Good job security	No	Ref.	Ref.	Ref.	Ref.	Ref.
	Yes	1.016 (0.089,1.944) *	0.778 (0.084,1.471) *	0.872 (-0.251,1.996)	-0.11 (-1.052,0.831)	-0.005 (-0.021,0.011)

### PhenoAge and GrimaAge AA

PhenoAgeAA was associated with job strain, active work- compared to reference low strain- in both unadjusted (β=-1.201 95%CI -2.292, -0.11) and adjusted (β=-1.301 95%CI -2.391, -0.212)) models.

In the unadjusted models, GrimAgeAA was associated with several exposures among which unemployment increase of more than 1.5 years β=1.741 (0.347,3.135) compared to employed, belonging to the white-collar occupational group decreases the aging of almost 1.5 years (β=-1.447, 95%CI -2.127, -0.768) and working for the state/municipality as well slowed the aging similarly (β=-1.219, 95%CI -1.885, -0.552). However, upon adjustment, only working as white-collar remained statistically significant a little above six months (β=-0.683, 95%CI -1.264, -0.102) when compared to blue collars.

### Rate of aging: DunedinPoAm

For the unadjusted fit, we found a statistically significant association for being working as white-collar - β=-0.015, 95%CI -0.026, -0.003) compared with being blue-collar. Effort (β=0.009, 95%CI 0.001,0.018) and evening shift increase the rate of aging of less than 2% (β=0.015, 95%CI 0.001,0.03). No statistically significant association was found once we adjusted for all the known risk factors.

### Sensitivity analysis: sex-stratified

Adjusted models were fitted for women and men separately. Among women (Supplementary Table 6 in Supplementary Materials), we found that being in a temporary job versus a permanent one presented a negative association with Horvath age acceleration (β=-1.484 95%CI -2.922, -0.047), whereas the estimate obtained for being continuously employed versus discontinuously with GrimAge (β= -0.696, 95%CI -1.309, -0.009) decreased the AA.

In contrast to our expectations, higher Effort-Reward Imbalance was associated with a decrease by approximately one year in the AA for both Horvath (β=-1.292, 95%CI -2.486, -0.099) and Hannum markers (β= -0.915, 95%CI -1.812, -0.019). It is worth underlying that if the effort-reward imbalance has a value below or close to zero, it indicates a favourable condition (low effort, high reward), whereas values above 1.0 indicate exposure to work stress, according to the model.

Long working hours and strenuous occupational physical activity (OPA) increased the AA of these markers by more than 2 years: for working more than 40 hours/week versus <31 hours/week we observed increases in Horvath AA (β=2.075, 95%CI 0.534, 3.616) and Hannum AA (β=2.483, 95%CI 0.421, 4.546), and for high-intensity strenuous physical effort and increase in Hannum AA (β=1.248 95%CI 0.088,2408).

For men (Supplementary Table 7 in Supplementary Materials), DunedinPoAm showed a decreased rate in job demand (β=-0.012, 95%CI -0.023, -0.001), but no effect was found for job control. An increase was observed for evening jobs compared with day ones (β=0.021, 95%CI 0.002, 0.039). Nevertheless, unlike observed among women, no significant effects were found for working hours in any of the outcomes analyzed.

Occupational physical activity seems to be beneficial in men, where high-intensity strenuous physical effort decreases AA for Horvath (β=-1.775, 95%CI -3.282, -0.267) and Hannum (β=-1.22, 95%CI -2.348, -0.092) with an opposite effect for GrimaAge in intermediate intensity (β=1.651 95%CI 0.4, 2.901). GrimAge was also negatively associated with white-collar occupational class (β=-1.245, 95%CI -2.164, -0.325). Lack of job security increased by more than one year the Hannum marker (β=1.132, 95%CI 2.191, 0.072), although it was expected to capture a stressful condition.

## DISCUSSION

In this work, we assessed the association (and its magnitude) of five biomarkers of epigenetic age acceleration with work-related stress and well-being indicators (as well as other employment characteristics) in the Northern Finland Birth Cohort 1966, at 46 years old. Overall, we have observed small magnitude of age acceleration for job-related stress variables in the range of ±2 years, compared with those of non-communicable disease risk factors (e.g. current smoking is associated with 4.73 years increasing GrimAge age acceleration (AA) marker).

Pooled linear regression results (men and women jointly) showed inconsistent patterns of associations of job stress compared with the current literature, and few statistically significant results once we adjusted for covariates. Horvath and Hannum EAA were positively (accelerating aging) associated with working longer hours, with a significant trend in risk, but in contrast to our expectations, were also positively associated with job security and negatively (decelerating aging) associated with higher effort, high strain and high effort-reward imbalance, although for the latter two differences were not significant. The other biomarkers were statistically significantly associated for job strain (decelerated epigenetic aging in active workers) for PhenoAgeAA and job category (decelerated EEA in white collars compared with blue collars in GrimAgeAA).

Once we stratified analyses by sex, a different pattern of association emerged, with women leading on the statistically significant results. Although job strain was overall not significantly associated with EAA, for some aging biomarkers, the effect size for women and men were in opposite directions. For example, in GrimAgeAA, women showed an accelerating effect for job demands and consequently for two job strain formulations. Among women, effort and effort-reward imbalance presented a less accelerating effect when compared with men, and the same pattern was detected with overcommitment for all the outcomes.

Long working hours(>55hours/week) and shift work have been associated with increased risk of chronic conditions like stroke or breast cancer [41, 42], and working long hours is a risk factor for shortened sleeping hours and difficulty falling asleep [43]. A Japanese study conducted among white-collar factory workers found that long working hours lead to sleep problems in a dose–response manner and impeded adequate recovery from fatigue, resulting in cumulative fatigue [44]. A previous study [45] found that men working long hours showed a worse cardiometabolic and inflammatory profile and increased anthropometric markers compared to those who did not work long hours; this was not confirmed in women, where these relations were absent or weak. Our study found that women have an increase of age acceleration over two years for Hannum and Horvath AA, when working for more than 40 hours/week. While we are not able to understand the biological mechanism, a study in UK found that among women working long hours and weekends deteriorates their mental health and increases depressive symptoms [46]. We hypothesize that mental health is acting as a mediator between the long hours of work and age acceleration, as findings of association between depression and AA start to emerge [47, 48].

High-intensity physical effort at work had an increasing effect for women but a decreasing effect in men. This contrasting result in men and women seems to point to the idea that the effects of job stress are different in the two genders [49], with women being affected more at an emotional level. While our intention was not to assess gender differences, nor we detect any substantial pattern in this sense, other fields such as occupational psychology [50] have assessed differences in work stress management. It has been observed that women express greater psychosomatic complaints when working in high demand, low control, and low support settings than their males [50]. While we have no evidence from our data, the increased effect in OPA seen for women might also be related to the heavy features of their jobs that like cleaning or nursing.

The effort-reward imbalance that represents “the interaction between a person’s cognitions, emotions, and behaviours, and the material and social work context” decreased the AA for both Horvath and Hannum markers in both genders, although significantly only among women. This seems in contrast with results from another study suggesting that women who experienced a higher level of reward showed more positive health functioning. This might be because women seem to experience a higher buffering effect from social support than from job control. Likewise, a recent study [51] conducted on the German socio-economic panel data, reported higher values for men in ‘effort’ and effort-reward ratio but no significant gender differences for the association between effort–reward imbalance model and the risk of self-reported depression.

Different epigenetic age acceleration indicators seem to represent different aspects of aging. HannumAA has been described as a biomarker of immune system ageing and demonstrated sensitivity to variations in the environment and lifestyle. At the same time, HorvathAA is a stable indicator of metabolic aging processes [52]. However, we have found that HorvathAA and HannumAA indicators are highly correlated, as reported by Lau et al. [53], in contrast with others who reported low correlation values [54]. Our results confirmed that the GrimAge clock is higher in smokers (past and current) and in relation to alcohol intake, as also found by Kresovich et al. [54]; in fact, the clock is constructed as the composite of 8 DNA methylation-based markers for plasma proteins and self-reported smoking packs [32] making it more responsive to smokers. Differences by gender were statistically significant for all AA markers, but female gender was positively associated with Horvath, Hannum and GrimAge, and negatively associated with PhenoAge and DunedinPoAm. This points to the observed difference between women and men, who present a diverse pattern in terms of epigenetics [49–51], which was the main reason to add, despite the small sample size, the sex-stratified analysis. Singmann et al. [55] have identified and validated 1,184 CpG sites to be differentially methylated between men and women; for these CpGs there is large overlap with the CpG sites used to define the EEAA. For Hannum the overlap is 28%, for Horvath 17%, for PhenoAge 14%, and for DunedinPoAm is 76%. However, the pace of aging is the indicator that discriminates less across variables.

Lastly, we observed that adjusted associations for the biomarkers with known risk factors are mostly not associated, except for smoking levels in GriamAgeAA and DunedinPoAm. While others have found similar pattern, for example, in Fiorito et al. [9], the effect size of the association of BMI, alcohol consumption, and physical activity was less than one-year comparing extreme categories and similar results have been obtained [56, 57].

This paper is one of the first attempts to address the working dimension of epigenetic age acceleration indicators, to the best of our knowledge. The NFBC 1966 cohort at the age of 46 years offers a rich questionnaire that allows studying a general population-based sample representing all occupations and sectors of economy which makes it an ideal setting of studying employment-related factors in relation to other health predictors. With this study population, where all the participants were born in the same year in the same geographical region, we minimized confounding by changes in working life circumstances along with macroeconomic trends. In other studies [9], the participants usually have different ages, geographical backgrounds, and working life exposures. As pointed out by Belsky et al. [33], the four age acceleration indicators were developed on blood DNA methylation, making them highly sensitive to changes in chronological age. The drawback is that '…clocks confound methylation patterns arising from early-life exposures to methylation-altering factors with methylation patterns related to biological ageing during adulthood' [33].

There are some limitations in this study. The limited sample size of subjects with both DNA methylation data and job variables affected the regression analysis. It could explain the lack of power in identifying robust work-stress associations, as also did the low Cronbach alpha for overcommitment and reward. Although the questionnaire is detailed on work-related factors, established work stress-related scales, details on job typologies, duration of the work stress or financial job insecurity have not been specifically investigated. Job strain was queried on a reduced number of items from the original Job Content Questionnaire [58], accounting for 42 items. Furthermore, due to the small sample size, other work-related indicators have been collapsed in binary variables, implying low variability.

The subjects in NFBC are mostly permanently occupied, with little worries about job security as 85% rated their job as secure. 77% worked less than or up to 40 hours per week and in a diurnal job with no shifts. Overall, the sample is homogenous, and stems from a Nordic welfare society with rather favourable working conditions and women participating in working life equally often as men, although the distribution of occupations is quite gendered as in most societies – women predominate nursing, men construction etc. Nevertheless, the characteristics of the NFBC dataset could be the reason for the inconsistent results observed. In a previous study conducted on the NFBC data (n=6496) Ek et al. [38] evaluated the employment trajectories over 30 years (ages 16 to 45), derived by latent class analysis of retrospective employment history calendars. It emerged that the employment trajectories most favourable for work-related well-being in midlife were rooted in social investments during early life and characterized by attainment of higher education and self-employment.

Nonetheless, this is one of the first studies to quantify the relation between a large variety of job-related variables and epigenetic age acceleration and pace of ageing. Our results suggest that women and men present different associations with different epigenetic distributions regarding work-related stress indicators. We advocate for further studies to be carried out for detailed patterns in different types of jobs [59] and in different societies as well as using measurements that target the longitudinal effects of the work environment and employment histories on stress and health and that account for gender differences.

## MATERIALS AND METHODS

### Study population

The study sample consisted of 604 participants from the ongoing Northern Finland Birth Cohort 1966 (NFBC), a longitudinal research program established to promote population-level health and well-being. The NFBC was started as a cohort of mothers and newborns with expected date in 1966 in the provinces of Oulu and Lapland (Finland), including over 95% of births in the region. The initial aim was to examine the risk factors in preterm birth and the consequences of adverse outcomes and subsequent morbidity. Later on, data were collected at 1, 14, 31 and 46 years old through clinical examination, questionnaires (lifestyle, employment and working conditions), and national records to improve population health and well-being [60, 61]. From the 46 years old questionnaires, we selected participants belonging to the work force [employed (part-time, full time, self-employed) and unemployed subjects] for which DNA methylation data [62] were available (a random sample), as shown in the flowchart (see Supplementary Figure 1 in Supplementary Materials).

### Computation of epigenetic clock measures

We have calculated four epigenetic age indicators: Horvath [1] DNAm age based on the weighted average of 353 age-related CpG; Hannum [6] DNAm age based on 71 blood specific age-related CpGs; Pheno [63] DNAm age based on 513 phenotypic age-related CpGs and DNAm GrimAge [32].

Based on these epigenetic clocks we defined the extrinsic epigenetic age acceleration (EAA), as the primary outcome, obtained from the residual values of the linear regression of epigenetic age on chronological age. Positive values of EAA indicated faster biological aging, while negative values indicated decelerated aging.

This four EAAs measure how much ageing has occurred in an individual up to the point of measurement. To assess how fast the subject is ageing, we included DunedinPoAm [33] an indicator based on 46 CpGs that is trained on longitudinal data and express a rate of biological aging (compared with the Dunedin sample on which the measure was trained).

### Work-related indicators

We used questionnaire items at age 46 years to obtain the participants’ job characteristics, employment history, and work-related stress and well-being indicators (see Table 1 for definitions). We included the current employment status as permanent, temporary, and unemployed. The employer as 'state/municipality' or 'private employer' and the occupational group in two levels: 'blue collars' (service, sale staff, care staff, farmers, building, repairs, transport workers) and 'white collars' (directors, senior management, advisors and official's office workers and customer service).

Indicating work-related stress, Job strain [58] was included as a linear term, as the quotient of demands over control and as categorical at three (low, intermediate, and high strain) and four levels (high strain, active, passive, low strain) reference. As another work stress measure, effort-reward imbalance (ERI) [64] was computed as means of reward and effort scales and as a means ratio (effort/reward). Furthermore, overcommitment was the sum of six items of questions that investigated the extent of excessive investment to work.

We included strenuousness of occupational physical activity (OPA) (low, moderate, and high intensity) and employment history (Continuously vs discontinuously employed); excessive working hours and shift (less than 31 hours per week, 31-40 hours, and more than 40 hours) and when working hours occurred as shift by splitting the answers between 'day job (06-18)' and 'evening/night job'.

Regarding work-related well-being, we collected work favouring attitude as the sum of five items from Kahn and Wiener [65]; and work engagement using the Utrecht Work Engagement Scale [66]. Good job security (yes/no) was collected. Details on the definition of work-related indicators and their Cronbach Alpha are in Supplementary Materials.

### Covariates

As additional variables, we have included established lifestyle-related risk factors for poor health and accelerated ageing. Smoking was classified as: never, past and current smoker. Alcohol consumption was categorized as a non-drinker, moderate or heavy drinker, based on questions on how often and what type of drinks (wine, spirits, beer/cider).

Body Mass Index (BMI) is presented in three levels (optimal < 24.9, overweight 25-29.9, obese>=30); the educational level was classified as basic (< 9 years of school and no vocational education or only short course), secondary (vocational school or college degree and/or matriculation examination) or tertiary (polytechnic or university degree) [67]. Leisure-time physical (LPA) activity has been derived by a combination of questions that accounted for the type of activity (brisk/light) and duration and weekly frequency and summarised in three levels: inactive, moderately active, and very active/active [68]. Age was excluded because chronological age has zero correlation with age acceleration measures (by definition) and we defined sex from birth records.

### Statistical methods

We computed the descriptive statistics (mean and standard deviation) for all the continuous variables and frequency for categorical variables and the Pearson correlation for the four EEAA measures and DunedinPoAm. To evaluate the association of the epigenetic clocks and DunedinPoAm with job measures, we evaluated the association using the Chi-Square test for categorical exposure, Student t-test or Analysis of variance for continuous ones. Initially, to assess the effect of risk factors on the biomarkers, we fitted linear regression models for all the outcomes. Further linear models were fitted for unadjusted and fully adjusted for sex, alcohol consumption, smoking, BMI, educational levels, and physical activity. As a sensitivity analysis, we investigated fully adjusted models separated by sex. Results are reported as estimates, and 95% confidence intervals and as standardized estimates. Linear regression assumptions were assessed on residual.

### Compliance with ethical standards

All participants gave written informed consent in accordance with the Declaration of Helsinki 1975, as revised in 2000, at each stage of the study. The Ethics Committee approved the study of the Northern Ostrobothnia Hospital District.

### Data availability

NFBC data is available from the University of Oulu, Infrastructure for Population Studies. Permission to use the data can be applied for research purposes via electronic material request portal. In the use of data, we follow the EU general data protection regulation (679/2016) and Finnish Data Protection Act. The use of personal data is based on cohort participant’s written informed consent at his/her latest follow-up study, which may cause limitations to its use. Please, contact NFBC project center (NFBCprojectcenter@oulu.fi) and visit the cohort website (http://www.oulu.fi/nfbc) for more information.

## Supplementary Material

Supplementary Materials

Supplementary Figure 1

Supplementary Tables
