# Supplementary material for: Work-related stress and well-being in association with epigenetic age acceleration: A Northern Finland Birth Cohort 1966 Study
Source: Aging (Albany NY). 2022 Feb 2;14(3):1128–56. doi: 10.18632/aging.203872 (PMC8876924; doi:10.18632/aging.203872)
Supplement: Supplementary Materials [file aging-14-203872-s001.pdf]

SUPPLEMENTARY MATERIALS

Work-related stress and well-being in association with epigenetic age acceleration: A Northern Finland Birth Cohort 1966 Study

DNA data collection

Fasting blood samples were collected at follow-up of participants at ages 31 and 46 years and stored at −80° C for subsequent biomarker profiling DNA samples were obtained from 5,923 subjects from NFBC1966. In 2012, all individuals with a known address in Finland were sent postal questionnaires and an invitation for a clinical examination questionnaire and clinical data was collected for 5539 participants. DNA methylation at 46 years was extracted for 807 randomly selected subjects with both questionnaire and clinical data. Among these individuals, DNA methylation data at 46 years were for 766 subjects. Detailed and technical aspects are reported [1].

Work-related indicators' definitions

Job strain

We computed the job strain dimension using a modified Karasek's Job Content Questionnaire [2]. Job control was measured using 9 items (Cronbach α =0. 86) and questions were rated from 'very little' (1) to 'a lot' (5). Job Control contains 3 items on the ability to use skills and learning new things and 6 items on the possibilities to decision making: influencing work tasks, order of performing tasks, work pace, methods, distribution, and with whom to work.

Job demands were measured using 4 items (Cronbach α =0. 81), investigating to what extent the following capabilities are required in performing own work: Reacting quickly, Paying attention to details, Patience, and Concentration. They were rated from' almost not at all' (1) to 'very much' (5). We first averaged the scores for control and demands items and analysed them separately, and then constructed 4 alternative formulations of job strain that have previously been used in studies on the demand-control model [3]:

i. The linear term was a continuous job strain variable obtained from the following equation: (0.5 x job demand score) - (0.5 x job control score).

ii. The categories were classified as "high strain" (high demands and low job control), "active" (high demands and high control), "passive" (low demands and low control), and "low strain" (low demands and high control), dividing demand and control at their median score [4].

iii. The quotient term was formed by dividing the sum of job demands by the sum of job control.

iv. The tertile job strain divided the distributions of demands and job control into thirds, as in a previous study by Green and Johnson [5]. The highest 2 tertiles in demands combined with the lowest 2 tertiles in job control formed the high-strain category. The lowest 2 tertiles in demands combined with the highest 2 tertiles in control formed the low-strain category. All other combinations were placed into the intermediate strain category. Job strain was coded as an ordinal variable ranging from low, intermediate, and high strain.

Effort-reward imbalance (ERI)

We measured the Effort-Reward imbalance (ERI) using the Occupational Stress Questionnaire [6]. Effort was measured with a three-item scale (e.g., 'I have constant time pressure due to a heavy workload') (Cronbach's α = 0.69) and reward with a four-item scale for reward (e.g., 'I receive the respect I deserve from my superiors') (α = 0.05), all rated on a scale from 1 (strongly agree) to 4 (strongly disagree). The Effort scale measures employees' time and energy invested in the job. The Reward scale, in turn, assesses contentment with the amount of salary, job security and self-acceptance at work. ERI was calculated by dividing the mean score of effort by the mean score of reward. That is, struggling with high efforts but receiving low rewards is postulated to increase the risk for experiencing work stress [7]. Hence, high scores of the ratio (effort/ reward) refer to higher job stress.

Overcommitment

We assessed overcommitment [6] using the sum of 6 items (Cronbach α =0. 43) rated from 'totally agree' (1) to 'totally disagree' (4). Questions investigated agreement with the statements of feeling easily paralyzed by the time pressures of the work; starting to think about work as soon as waking up in the morning; finding it easy to relax and "switch off" when coming home from work; the close ones telling me that I sacrifice too much to my work; being rarely able to let go of work stuff which stays on my mind when I go to bed; and having it difficult to fall asleep when postponing something that was due today.

Occupational physical activity (OPA)

Levels of physical job strenuousness were evaluated using the question "to what extent are the following

tasks and postures part of your job." The participants had to evaluate the extent of certain tasks (e.g., "heavy physical work in which the body has to struggle," "lifting loads over 15 kg") and postures (e.g., "standing," "bending") in their work. The response scale was from 'not at all or very rarely' (1) to 'very often' (5). We summed and divided the scores into three groups on the basis of the tertile cut-offs: low, moderate, and high intensity [8].

Employment history

From the questionnaire, we selected the question [9]: which of the following alternatives (from always continuously employed to never in gainful employment) best describes your employment history? We created two groups: the' Continuously employed' including those who answered, 'I have always been employed through my work history' and the 'at least temporary unemployed' including those who answered to have experienced occasional unemployment, more time as unemployed than as employed or never have done paid work.

Working hours and shift

From the questionnaire, we selected the question 'how many hours of paid work do you do on average per week?' and we categorized in three levels: less than 31 hours per week, 31-40 hours, and more than 40 hours. And to assess when those working hours occurred, we split the answers between 'day job (06-18)' and 'evening/shift job'.

Work-favouring attitude

Reflecting personal attitude and commitment towards one's work-related social role, work favouring attitude was measured using the scale introduced by Kahn and Wiener [10]. Of the original six items, five represent positive work-favouring attitudes (e.g., "Work, for me, is a calling, or a way to exercise and master gratifying skills, or a means to provide income") and one item represents a negative attitude ("Work is a necessary evil one has to do to make a living"), that was excluded (Cronbach α =0. 79). In remaining 5-item scale was used indicating the magnitude of a positive work-favouring attitude, answers ranging from 'very little' (1) to 'very much' (5) [11].

Work engagement

Work engagement is obtained as the sum of the score of 9 items (Cronbach α =0. 92) using the Utrecht Work Engagement Scale [12]. We used the mean score [11] of nine items measured on a 7-point Likert-scale (e.g., "I feel bursting with energy when I am working") asking how often the item could be endorsed by the participant (ranging from 0= never to 6=everyday).

Job security

We assessed with one question the perception of job security and collapsed the four possible answers from 'very good' (1) to 'very poor' (4), in two levels 'Yes' (1 and 2) and 'No' (3 and 4).

REFERENCES

1. Sovio U, Bennett AJ, Millwood IY, Molitor J, O'Reilly PF, Timpson NJ, Kaakinen M, Laitinen J, Haukka J, Pillas D, Tzoulaki I, Molitor J, Hoggart C, et al. Genetic determinants of height growth assessed longitudinally from infancy to adulthood in the northern Finland birth cohort 1966. PLoS Genet. 2009; 5:e1000409. https://doi.org/10.1371/journal.pgen.1000409 PMID:19266077

2. Karasek R, Brisson C, Kawakami N, Houtman I, Bongers P, Amick B. The Job Content Questionnaire (JCQ): an instrument for internationally comparative assessments of psychosocial job characteristics. J Occup Health Psychol. 1998; 3:322–55. https://doi.org/10.1037//1076-8998.3.4.322 PMID:9805280

3. Hintsanen M, Kivimäki M, Elovainio M, Pulkki-Råback L, Keskivaara P, Juonala M, Raitakari OT, Keltikangas-Järvinen L. Job strain and early atherosclerosis: the Cardiovascular Risk in Young Finns study. Psychosom Med. 2005; 67:740–7. https://doi.org/10.1097/01.psy.0000181271.04169.93 PMID:16204432

4. Kujala V, Tammelin T, Remes J, Vammavaara E, Ek E, Laitinen J. Work ability index of young employees and their sickness absence during the following year. Scand J Work Environ Health. 2006; 32:75–84. https://doi.org/10.5271/sjweh.979 PMID:16539175

5. Green KL, Johnson JV. The effects of psychosocial work organization on patterns of cigarette smoking among male chemical plant employees. Am J Public Health. 1990; 80:1368–71. https://doi.org/10.2105/ajph.80.11.1368 PMID:2240307

6. Siegrist J, Starke D, Chandola T, Godin I, Marmot M, Niedhammer I, Peter R. The measurement of effort-reward imbalance at work: European comparisons. Soc Sci Med. 2004; 58:1483–99. https://doi.org/10.1016/S0277-9536(03)00351-4 PMID:14759692

7. Siegrist J. Adverse health effects of high-effort/low-reward conditions. J Occup Health Psychol. 1996; 1:27–41.

https://doi.org/10.1037//1076-8998.1.1.27
PMID:9547031

8. Nevanperä N, Seitsamo J, Ala-Mursula L, Remes J, Hopsu L, Auvinen J, Tammelin T, Järvelin MR, Laitinen J. Perceived Work Ability in the Light of Long-Term and Stress-Related Unhealthy Behaviors-a Prospective Cohort Study. Int J Behav Med. 2016; 23:179–89.
https://doi.org/10.1007/s12529-015-9512-0
PMID:26500090

9. Punakallio A, Lusa S, Ala-Mursula L, Ek E, Nevanperä N, Remes J, Auvinen J, Seitsamo J, Karppinen J, Laitinen J. Personal meaning of work and perceived work ability among middle-aged workers with physically strenuous work: a Northern Finland Birth Cohort 1966 Study. Int Arch Occup Environ Health. 2019; 92:371–81.
https://doi.org/10.1007/s00420-019-01412-9
PMID:30767053

10. Kahn H, Wiener AJ. year 2000; a framework for speculation on the next thirty-three years. 1967. FAO of UN https://agris.fao.org/agris-search/search.do?recordID=US201300314687

11. Ek E, Ala-Mursula L, Velázquez RG, Tolvanen A, Salmela-Aro K. Employment trajectories until midlife associate with early social role investments and current work-related well-being. Advances in Life Course Research. 2021; 47:100391.
https://doi.org/10.1016/j.alcr.2020.100391

12. Schaufeli W, Salanova M, González-Romà, Bakker, A. The measurement of Engagement and Burnout: A two sample confirmatory factor anañytic approach. 2008:71–92.
https://doi.org/10.1023/A:1015630930326
