# Supplementary figures and images for: Work-related stress and well-being in association with epigenetic age acceleration: A Northern Finland Birth Cohort 1966 Study
Source: Aging (Albany NY). 2022 Feb 2;14(3):1128–56. doi: 10.18632/aging.203872 (PMC8876924; doi:10.18632/aging.203872)

## SUPPLEMENTARY FIGURE

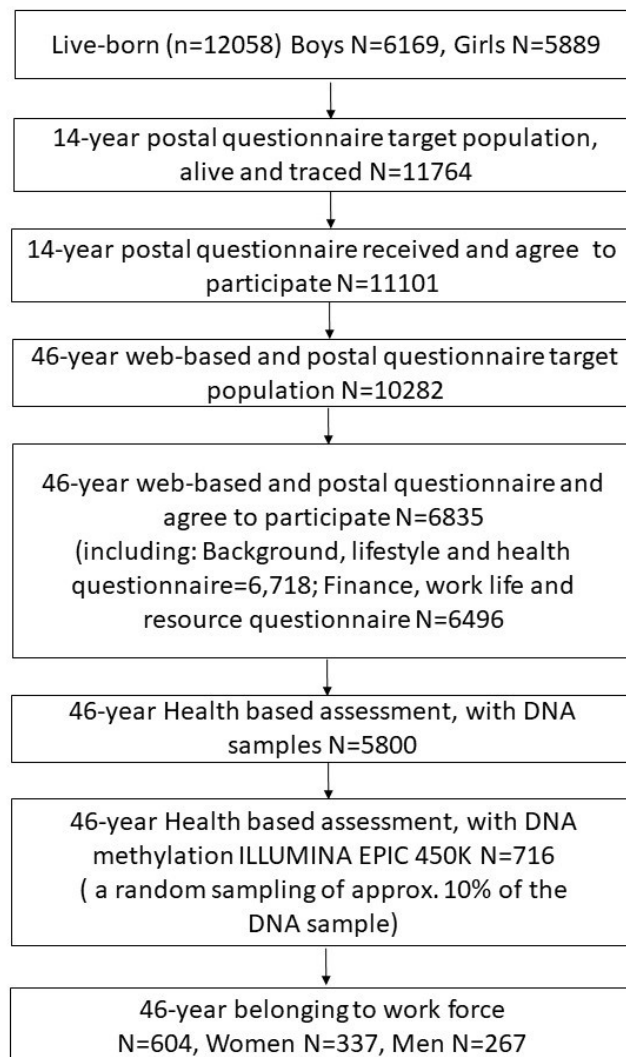

Supplementary Figure 1. Flowchart of the study population in the NFBC 1966.

Supplement: Supplementary Figure 1 [file aging-14-203872-s002.pdf]
