# Supplementary material for: Work-related stress and well-being in association with epigenetic age acceleration: A Northern Finland Birth Cohort 1966 Study
Source: Aging (Albany NY). 2022 Feb 2;14(3):1128–56. doi: 10.18632/aging.203872 (PMC8876924; doi:10.18632/aging.203872)
Supplement: Supplementary Tables [file aging-14-203872-s003.pdf]

SUPPLEMENTARY TABLES

Supplementary Table 1. P-values for analysis of variance (ANOVA) and chi-square test, for all job exposures and risk factors.

**ANOVA P-values**	**Sex***	**BMI**	**Education**	**Physical activity**	**Alcohol consumption**	**Smoking**
Control	0.04	0.18	<0.001	0.34	0.82	0.65
Demand	<0.001	0.31	<0.001	0.24	0.12	0.38
Job strain	<0.001	0.45	<0.001	0.57	0.32	0.45
Job strain linear	<0.001	0.33	<0.001	0.37	0.23	0.5
Job strain quotient	<0.001	0.45	<0.001	0.57	0.32	0.45
Effort	0.01	0.62	<0.001	0.47	0.61	<0.001
Reward	0.29	0.56	0.03	0.91	<0.001	0.22
Effort-Reward Imbalance	0.03	0.9	0.2	0.58	0.24	0.02
Overcommitment	0.02	0.35	0.05	0.08	0.91	0.09
Work attitude	<0.001	0.54	<0.001	0.1	0.2	0.24
Work engagement	<0.001	0.49	<0.001	0.06	0.05	0.1
**Chi Square P-values**						
Job status	<0.001	0.61	0.27	0.86	0.71	0.23
Employer	<0.001	0.17	<0.001	0.57	0.03	<0.001
Occupational group	0.98	0.81	<0.001	0.86	0.3	<0.001
Job Strain quartile	<0.001	0.3	<0.001	0.89	0.28	0.75
Job Strain Revised	<0.001	0.05	<0.001	0.16	0.07	0.71
Work history	0.26	0.38	0.05	0.86	0.76	0.2
OPA	0.21	0.05	<0.001	0.71	0.55	0.06
Working hours	<0.001	0.87	0.04	0.12	<0.001	0.36
Shift	0.53	0.14	<0.001	0.66	0.05	0.03
Job Security	0.84	0.7	0.91	0.95	0.19	0.13

*For sex is a t-test as it has only two levels.

Supplementary Table 2. Pearson correlations among extrinsic epigenetic age acceleration and pace of ageing.

**N=604**	**Horvath**	**Hannum**	**PhenoAge**	**GrimAge**
Horvath				
Hannum	0.96			
PhenoAge	0.48	0.47		
GrimAge	0.23	0.20	0.31	
DunedinPoAm	0.24	020	0.33	0.78

Supplementary Table 3. Adjusted coefficient estimates from linear regression with 95% confidence intervals.

		HorvathAA Estimates (95% CI)	HannumAA Estimates (95% CI)	PhenoAgeAA Estimates (95% CI)	GrimAgeAA Estimates (95% CI)	DunedinPoAm Estimates (95% CI)
Job Status	permanent	Ref.	Ref.	Ref.	Ref.	Ref.
	temporary	-1.127 (-2.422,0.168)	-0.694 (-1.661,0.272)	-0.505 (-2.017,1.008)	0.631 (-0.341,1.602)	0.005 (-0.013,0.023)
	unemployed	0.063 (-1.325,1.451)	-0.006 (-1.042,1.03)	0.817 (-0.804,2.438)	0.602 (-0.439,1.644)	0.004 (-0.015,0.023)
Employer	private employer	Ref.	Ref.	Ref.	Ref.	Ref.
	state/municipality	0.063 (-0.699,0.825)	0.048 (-0.522,0.618)	0.116 (-0.775,1.008)	-0.096 (-0.655,0.463)	0 (-0.011,0.01)
Occupational group	Blue collars	Ref.	Ref.	Ref.	Ref.	Ref.
	White collars	0.509 (-0.274,1.292)	0.318 (-0.268,0.905)	0.257 (-0.653,1.166)	-0.683 (-1.264,-0.102) *	-0.002 (-0.013,0.008)
Job control		0.317 (-0.149,0.782)	0.169 (-0.179,0.517)	-0.186 (-0.73,0.359)	-0.154 (-0.508,0.199)	-0.001 (-0.007,0.006)
Job demand		0.086 (-0.446,0.618)	0.118 (-0.279,0.514)	-0.483 (-1.103,0.137)	-0.018 (-0.42,0.384)	-0.004 (-0.012,0.003)
Job strain Linear		-0.41 (-1.234,0.415)	-0.154 (-0.769,0.462)	-0.354 (-1.314,0.605)	0.157 (-0.465,0.779)	-0.005 (-0.017,0.006)
Job strain	Low strain	Ref.	Ref.	Ref.	Ref.	Ref.
	active work	0.217 (-0.724,1.158)	0.271 (-0.431,0.973)	-1.301 (-2.391,-0.212) *	0.254 (-0.454,0.963)	-0.005 (-0.018,0.009)
	passive work	0.177 (-0.856,1.21)	0.223 (-0.547,0.994)	-0.666 (-1.862,0.53)	0.518 (-0.26,1.296)	0.005 (-0.01,0.019)
	high strain	-0.189 (-1.256,0.877)	-0.037 (-0.832,0.759)	-0.527 (-1.761,0.708)	0.526 (-0.277,1.329)	0 (-0.015,0.015)
Job strain quotient		-0.843 (-2.066,0.379)	-0.414 (-1.328,0.499)	-0.568 (-1.992,0.855)	0.234 (-0.69,1.157)	-0.009 (-0.026,0.008)
Job strain tertile	Low strain	Ref.	Ref.	Ref.	Ref.	Ref.
	Intermediate strain	-0.328 (-1.194,0.538)	-0.101 (-0.748,0.545)	-0.942 (-1.946,0.062)	0.31 (-0.343,0.963)	0.001 (-0.012,0.013)
	high strain	-0.226 (-1.109,0.657)	-0.032 (-0.691,0.627)	-0.283 (-1.307,0.741)	0.133 (-0.532,0.799)	-0.002 (-0.014,0.011)
Effort		-0.582 (-1.098,-0.066) *	-0.44 (-0.825,-0.054) *	-0.323 (-0.932,0.285)	0.013 (-0.381,0.408)	0.001 (-0.006,0.008)
Reward		0.115 (-0.53,0.76)	0.212 (-0.27,0.693)	0.049 (-0.705,0.803)	0.095 (-0.391,0.58)	-0.005 (-0.014,0.004)
Effort-Reward Imbalance		-0.734 (-1.598,0.13)	-0.61 (-1.255,0.036)	-0.38 (-1.394,0.634)	0.021 (-0.632,0.673)	0.004 (-0.008,0.016)
Overcommitment		0.097 (-0.448,0.641)	0.065 (-0.343,0.472)	0.24 (-0.396,0.876)	-0.029 (-0.443,0.386)	0 (-0.008,0.008)
Work history	At least temporary unemployed	Ref.	Ref.	Ref.	Ref.	Ref.
	Continuously Employed	0.205 (-0.46,0.87)	0.232 (-0.263,0.728)	-0.064 (-0.838,0.709)	-0.406 (-0.905,0.092)	-0.005 (-0.014,0.005)
Occupational Physical Activity strenuousness	Low Intensity	Ref.	Ref.	Ref.	Ref.	Ref.
	Intermediate Intensity	-0.357 (-1.42,0.705)	-0.176 (-0.971,0.618)	0.472 (-0.775,1.719)	0.638 (-0.169,1.445)	0.002 (-0.013,0.017)
	High Intensity	-0.345 (-1.406,0.715)	-0.087 (-0.88,0.705)	0.623 (-0.621,1.867)	0.017 (-0.787,0.822)	-0.013 (-0.028,0.002)
Working hours per week	less than 31 hours	Ref.	Ref.	Ref.	Ref.	Ref.

	31-40 hours	1.511 (-0.001,3.022) *	1.171 (0.042,2.3) *	-0.446 (-2.227,1.336)	-0.629 (-1.783,0.526)	-0.006 (-0.028,0.015)
	more than 40 hours	2.009 (0.368,3.651) *	1.547 (0.321,2.772) *	0.197 (-1.737,2.132)	-0.657 (-1.911,0.597)	-0.005 (-0.028,0.018)
Working shift	Day job	Ref.	Ref.	Ref.	Ref.	Ref.
	Evening/shift	0.346 (-0.54,1.232)	0.342 (-0.322,1.005)	0.481 (-0.562,1.524)	0.176 (-0.5,0.852)	0.007 (-0.005,0.02)
Work attitude		0.025 (-0.073,0.123)	0.01 (-0.063,0.083)	-0.006 (-0.12,0.109)	0.04 (-0.033,0.112)	0 (-0.002,0.001)
Work engagement		0.012 (-0.022,0.046)	0.008 (-0.018,0.034)	0.01 (-0.03,0.05)	-0.01 (-0.036,0.016)	0 (-0.001,0)
Job security	No	Ref.	Ref.	Ref.	Ref.	Ref.
	Yes	1.115 (0.16,2.071) *	0.841 (0.127,1.555) *	1.015 (-0.109,2.138)	-0.01 (-0.74,0.72)	-0.004 (-0.018,0.009)

Fully adjusted models for: BMI, alcohol consumption, educational level, leisure-time physical activity and smoking.
*Relevant confidence intervals.

Supplementary Table 4. Standardized unadjusted coefficient estimates from linear regression with 95% confidence intervals.

		HorvathAA	HannumAA	PhenoAgeAA	GrimAgeAA	DunedinPoAm
		Estimates (95% CI)	Estimates (95% CI)	Estimates (95% CI)	Estimates (95% CI)	Estimates (95% CI)
Job Status	permanent	Ref.	Ref.	Ref.	Ref.	Ref.
	temporary	-0.319 (-0.63,-0.008)*	-0.275 (-0.586,0.036)	0.027 (-0.284,0.339)	0.149 (-0.162,0.459)	0.202 (-0.109,0.513)
	unemployed	0.053 (-0.29,0.395)	0.035 (-0.308,0.378)	0.186 (-0.157,0.529)	0.427 (0.085,0.768)*	0.27 (-0.072,0.613)
Employer	private employer	Ref.	Ref.	Ref.	Ref.	Ref.
	state/municipality	-0.085 (-0.253,0.083)	-0.085 (-0.253,0.083)	0.039 (-0.129,0.207)	-0.299 (-0.462,-0.135)	-0.079 (-0.245,0.086)
Occupational group	Blue collars	Ref.	Ref.	Ref.	Ref.	Ref.
	White collars	0.034 (-0.137,0.205)	0.03 (-0.142,0.201)	0.001 (-0.17,0.172)	-0.355 (-0.521,-0.188)*	-0.208 (-0.374,-0.042)*
Job control		0.082 (-0.027,0.191)	0.066 (-0.043,0.176)	-0.047 (-0.156,0.062)	-0.055 (-0.163,0.052)	-0.062 (-0.171,0.046)
Job demand		0.006 (-0.119,0.13)	0.016 (-0.109,0.141)	-0.064 (-0.189,0.061)	-0.087 (-0.21,0.036)	-0.066 (-0.189,0.058)
Job strain Linear		-0.122 (-0.314,0.07)	-0.09 (-0.282,0.102)	-0.02 (-0.211,0.171)	-0.037 (-0.226,0.151)	-0.006 (-0.195,0.184)
Job strain	Low strain	Ref.	Ref.	Ref.	Ref.	Ref.
	active work	0.038 (-0.191,0.267)	0.057 (-0.173,0.286)	-0.25 (-0.477,-0.023)*	-0.084 (-0.308,0.14)	-0.116 (-0.342,0.109)
	passive work	0.028 (-0.221,0.277)	0.046 (-0.203,0.296)	-0.143 (-0.39,0.104)	0.052 (-0.192,0.295)	0.054 (-0.191,0.299)
	high strain	-0.089 (-0.337,0.16)	-0.075 (-0.324,0.174)	-0.055 (-0.301,0.192)	0.004 (-0.24,0.248)	0.071 (-0.173,0.316)
Job strain quotient		-0.234 (-0.521,0.053)	-0.186 (-0.474,0.101)	-0.048 (-0.334,0.238)	0.014 (-0.267,0.296)	0.011 (-0.272,0.294)
Job strain tertile	Low strain	Ref.	Ref.	Ref.	Ref.	Ref.
	Intermediate strain	-0.12 (-0.328,0.088)	-0.079 (-0.287,0.129)	-0.214 (-0.42,-0.007)*	0.01 (-0.194,0.214)	-0.002 (-0.207,0.203)
	high strain	-0.11 (-0.318,0.097)	-0.085 (-0.292,0.123)	-0.04 (-0.246,0.166)	-0.108 (-0.311,0.095)	-0.009 (-0.214,0.196)
Effort		-0.103 (-0.227,0.021)	-0.104 (-0.228,0.02)	-0.051 (-0.176,0.074)	0.186 (0.063,0.308)*	0.136 (0.012,0.26)*
Reward		0.022	0.059	0.049	0.08	0.03 (-0.122,0.183)

		(-0.132,0.176)	(-0.095,0.214)	(-0.105,0.204)	(-0.071,0.232)	
Effort-Reward Imbalance		-0.131 (-0.341,0.079)	-0.151 (-0.361,0.059)	-0.082 (-0.294,0.129)	0.195 (-0.012,0.401)	0.159 (-0.049,0.367)
Overcommitment		0.043 (-0.088,0.173)	0.034 (-0.097,0.165)	0.027 (-0.104,0.158)	0.036 (-0.094,0.166)	0 (-0.132,0.131)
Work history	At least temporary unemployed	Ref.	Ref.	Ref.	Ref.	Ref.
	Continuously Employed	0.059 (-0.102,0.22)	0.08 (-0.081,0.241)	-0.023 (-0.184,0.138)	-0.163 (-0.324,-0.002)*	-0.141 (-0.302,0.02)
Occupational Physical Activity	Low Intensity	Ref.	Ref.	Ref.	Ref.	Ref.
strenuousness	Intermediate Intensity	-0.07 (-0.325,0.185)	-0.05 (-0.305,0.206)	0.093 (-0.164,0.35)	0.335 (0.084,0.587)*	0.183 (-0.072,0.439)
	High Intensity	0.016 (-0.235,0.268)	0.053 (-0.199,0.305)	0.157 (-0.097,0.41)	0.166 (-0.082,0.415)	-0.048 (-0.3,0.204)
Working hours per week	less than 31 hours	Ref.	Ref.	Ref.	Ref.	Ref.
	31-40 hours	0.311 (-0.045,0.667)	0.321 (-0.036,0.677)	-0.108 (-0.469,0.253)	0.011 (-0.345,0.366)	-0.022 (-0.382,0.337)
	more than 40 hours	0.513 (0.129,0.898)*	0.524 (0.139,0.909)*	0.018 (-0.372,0.407)	0.14 (-0.243,0.524)	0.011 (-0.376,0.399)
Working shift	Day job	Ref.	Ref.	Ref.	Ref.	Ref.
	Evening/shift	0.113 (-0.095,0.322)	0.131 (-0.079,0.341)	0.122 (-0.089,0.333)	0.18 (-0.028,0.387)	0.217 (0.008,0.426)*
Work attitude		-0.001 (-0.024,0.022)	-0.003 (-0.026,0.02)	-0.001 (-0.024,0.022)	-0.01 (-0.033,0.013)	-0.013 (-0.036,0.009)
Work engagement		0.001 (-0.007,0.009)	0.001 (-0.007,0.009)	0.002 (-0.006,0.01)	-0.012 (-0.02,-0.004)*	-0.009 (-0.017,-0.001)*
Job security	No	Ref.	Ref.	Ref.	Ref.	Ref.
	Yes	0.254 (0.022,0.485)*	0.26 (0.028,0.492)*	0.182 (-0.052,0.416)	-0.027 (-0.258,0.204)	-0.072 (-0.305,0.16)

*Relevant confidence intervals.

Supplementary Table 5. Standardized adjusted linear regression coefficient estimates with 95% confidence interval for the epigenetic age and pace of aging.

		HorvathAA Estimates (95% CI)	HannumAA Estimates (95% CI)	PhenoAgeAA Estimates (95% CI)	GrimAgeAA Estimates (95% CI)	DunedinPoAm Estimates (95% CI)
Job Status	permanent	Ref.	Ref.	Ref.	Ref.	Ref.
	temporary	-1.127 (-2.422,0.168)	-0.694 (-1.661,0.272)	-0.505 (-2.017,1.008)	0.631 (-0.341,1.602)	0.005 (-0.013,0.023)
	unemployed	0.063 (-1.325,1.451)	-0.006 (-1.042,1.03)	0.817 (-0.804,2.438)	0.602 (-0.439,1.644)	0.004 (-0.015,0.023)
Employer	private employer	Ref.	Ref.	Ref.	Ref.	Ref.
	state/municipality	0.563 (-0.42,1.546)	0.297 (-0.441,1.034)	0.415 (-0.736,1.567)	0.25 (-0.454,0.953)	0.003 (-0.01,0.017)
Occupational group	Blue collars	Ref.	Ref.	Ref.	Ref.	Ref.
	White collars	-0.509 (-1.292,0.274)	-0.318 (-0.905,0.268)	-0.257 (-1.166,0.653)	0.683 (0.102,1.264)*	0.002 (-0.008,0.013)
Job control		0.317 (-0.149,0.782)	0.169 (-0.179,0.517)	-0.186 (-0.73,0.359)	-0.154 (-0.508,0.199)	-0.001 (-0.007,0.006)
Job demand		0.086 (-0.446,0.618)	0.118 (-0.279,0.514)	-0.483 (-1.103,0.137)	-0.018 (-0.42,0.384)	-0.004 (-0.012,0.003)
Job strain Linear		-0.41 (-1.234,0.415)	-0.154 (-0.769,0.462)	-0.354 (-1.314,0.605)	0.157 (-0.465,0.779)	-0.005 (-0.017,0.006)
Job strain	Low strain	Ref.	Ref.	Ref.	Ref.	Ref.

	active work	0.217 (-0.724,1.158)	0.271 (-0.431,0.973)	-1.301 (-2.391,-0.212)*	0.254 (-0.454,0.963)	-0.005 (-0.018,0.009)
	passive work	0.177 (-0.856,1.21)	0.223 (-0.547,0.994)	-0.666 (-1.862,0.53)	0.518 (-0.26,1.296)	0.005 (-0.01,0.019)
	high strain	-0.189 (-1.256,0.877)	-0.037 (-0.832,0.759)	-0.527 (-1.761,0.708)	0.526 (-0.277,1.329)	0 (-0.015,0.015)
Job strain quotient		-0.843 (-2.066,0.379)	-0.414 (-1.328,0.499)	-0.568 (-1.992,0.855)	0.234 (-0.69,1.157)	-0.009 (-0.026,0.008)
	Low strain	Ref.	Ref.	Ref.	Ref.	Ref.
Job strain tertile	Intermediate strain	-0.328 (-1.194,0.538)	-0.101 (-0.748,0.545)	-0.942 (-1.946,0.062)	0.31 (-0.343,0.963)	0.001 (-0.012,0.013)
	high strain	-0.226 (-1.109,0.657)	-0.032 (-0.691,0.627)	-0.283 (-1.307,0.741)	0.133 (-0.532,0.799)	-0.002 (-0.014,0.011)
Effort		-0.582 (-1.098,-0.066)*	-0.44 (-0.825,-0.054)*	-0.323 (-0.932,0.285)	0.013 (-0.381,0.408)	0.001 (-0.006,0.008)
Reward		0.115 (-0.53,0.76)	0.212 (-0.27,0.693)	0.049 (-0.705,0.803)	0.095 (-0.391,0.58)	-0.005 (-0.014,0.004)
Effort-Reward Imbalance		-0.734 (-1.598,0.13)	-0.61 (-1.255,0.036)	-0.38 (-1.394,0.634)	0.021 (-0.632,0.673)	0.004 (-0.008,0.016)
Overcommitment		0.097 (-0.448,0.641)	0.065 (-0.343,0.472)	0.24 (-0.396,0.876)	-0.029 (-0.443,0.386)	0 (-0.008,0.008)
Work history	At least temporary unemployed	Ref.	Ref.	Ref.	Ref.	Ref.
	Continuously Employed	0.205 (-0.46,0.87)	0.232 (-0.263,0.728)	-0.064 (-0.838,0.709)	-0.406 (-0.905,0.092)	-0.005 (-0.014,0.005)
Occupational Physical Activity	Low Intensity	Ref.	Ref.	Ref.	Ref.	Ref.
strenuousness	Intermediate Intensity	-0.357 (-1.42,0.705)	-0.176 (-0.971,0.618)	0.472 (-0.775,1.719)	0.638 (-0.169,1.445)	0.002 (-0.013,0.017)
	High Intensity	-0.345 (-1.406,0.715)	-0.087 (-0.88,0.705)	0.623 (-0.621,1.867)	0.017 (-0.787,0.822)	-0.013 (-0.028,0.002)
	less than 31 hours	Ref.	Ref.	Ref.	Ref.	Ref.
Working hours per week	31-40 hours	1.511 (-0.001,3.022)*	1.171 (0.042,2.3)*	-0.446 (-2.227,1.336)	-0.629 (-1.783,0.526)	-0.006 (-0.028,0.015)
	more than 40 hours	2.009 (0.368,3.651)*	1.547 (0.321,2.772)*	0.197 (-1.737,2.132)	-0.657 (-1.911,0.597)	-0.005 (-0.028,0.018)
Working shift	Day job	Ref.	Ref.	Ref.	Ref.	Ref.
	Evening/shift	0.346 (-0.54,1.232)	0.342 (-0.322,1.005)	0.481 (-0.562,1.524)	0.176 (-0.5,0.852)	0.007 (-0.005,0.02)
Work attitude		0.025 (-0.073,0.123)	0.01 (-0.063,0.083)	-0.006 (-0.12,0.109)	0.04 (-0.033,0.112)	0 (-0.002,0.001)
Work engagement		0.012 (-0.022,0.046)	0.008 (-0.018,0.034)	0.01 (-0.03,0.05)	-0.01 (-0.036,0.016)	0 (-0.001,0)
Job security	No	Ref.	Ref.	Ref.	Ref.	Ref.
	Yes	-1.115 (-2.071,-0.16)*	-0.841 (-1.555,-0.127)*	-1.015 (-2.138,0.109)	0.01 (-0.72,0.74)	0.004 (-0.009,0.018)

Fully adjusted models for: BMI, alcohol consumption, educational level, leisure-time physical activity and smoking.
*Relevant confidence intervals.

Supplementary Table 6. Women adjusted linear regression coefficient estimates with 95% confidence interval for the epigenetic age and pace of aging.

		HorvathAA	HannumAA	PhenoAgeAA	GrimAgeAA	DunedinPoAm
		Estimates (95% CI)	Estimates (95% CI)	Estimates (95% CI)	Estimates (95% CI)	Estimates (95% CI)
Job Status	permanent	Ref.	Ref.	Ref.	Ref.	Ref.
	temporary	-1.484 (-2.922,-0.047) *	-0.998 (-2.079,0.082)	-0.889 (-2.592,0.813)	0.762 (-0.298,1.822)	0.002 (-0.018,0.023)
	unemployed	0.012 (-2.01,2.034)	-0.128 (-1.648,1.392)	1.125 (-1.27,3.52)	1.463 (-0.028,2.955)	0.009 (-0.019,0.038)
Employer	private employer	Ref.	Ref.	Ref.	Ref.	Ref.
	state/municipality	0.563 (-0.42,1.546)	0.297 (-0.441,1.034)	0.415 (-0.736,1.567)	0.25 (-0.454,0.953)	0.003 (-0.01,0.017)
Occupational group	Blue collars	Ref.	Ref.	Ref.	Ref.	Ref.
	White collars	0.557 (-0.482,1.596)	0.447 (-0.332,1.225)	0.577 (-0.622,1.776)	-0.306 (-1.063,0.451)	0.007 (-0.007,0.022)
Job control		0.413 (-0.219,1.045)	0.297 (-0.177,0.77)	-0.079 (-0.818,0.659)	-0.285 (-0.749,0.178)	0.005 (-0.004,0.014)
Job demand		0.203 (-0.549,0.954)	0.262 (-0.299,0.824)	-0.377 (-1.251,0.498)	0.271 (-0.279,0.82)	0.002 (-0.008,0.013)
Job strain Linear		-0.398 (-1.485,0.689)	-0.185 (-0.999,0.629)	-0.435 (-1.694,0.824)	0.603 (-0.184,1.389)	-0.006 (-0.022,0.009)
Job strain	Low strain	Ref.	Ref.	Ref.	Ref.	Ref.
	active work	0.411 (-0.964,1.786)	0.393 (-0.634,1.421)	-1.414 (-3.006,0.179)	0.501 (-0.499,1.5)	0.004 (-0.015,0.023)
	passive work	0.38 (-1.156,1.917)	0.298 (-0.85,1.446)	-0.915 (-2.694,0.864)	0.316 (-0.801,1.433)	-0.003 (-0.025,0.019)
	high strain	-0.611 (-2.006,0.784)	-0.502 (-1.544,0.54)	-1.188 (-2.803,0.427)	0.754 (-0.26,1.768)	-0.003 (-0.022,0.017)
Job strain quotient		-1.009 (-2.579,0.561)	-0.525 (-1.701,0.652)	-0.727 (-2.549,1.094)	0.728 (-0.412,1.867)	-0.014 (-0.036,0.008)
Job strain tertile	Low strain	Ref.	Ref.	Ref.	Ref.	Ref.
	Intermediate strain	0.055 (-1.198,1.309)	0.198 (-0.74,1.136)	-0.958 (-2.407,0.491)	0.216 (-0.692,1.124)	-0.009 (-0.026,0.009)
	high strain	-0.21 (-1.397,0.977)	-0.148 (-1.036,0.74)	-0.567 (-1.939,0.805)	0.541 (-0.319,1.401)	-0.007 (-0.023,0.01)
Effort		-0.985 (-1.67,-0.3) *	-0.679 (-1.193,-0.165) *	-0.737 (-1.547,0.072)	-0.362 (-0.874,0.149)	-0.006 (-0.016,0.004)
Reward		-0.007 (-0.881,0.866)	0.06 (-0.596,0.715)	-0.223 (-1.243,0.796)	0.596 (-0.03,1.223)	-0.003 (-0.015,0.01)
Effort-Reward Imbalance		-1.292 (-2.486,-0.099) *	-0.915 (-1.812,-0.019) *	-0.669 (-2.075,0.737)	-0.769 (-1.635,0.096)	-0.007 (-0.024,0.009)
Overcommitment		-0.482 (-1.189,0.224)	-0.339 (-0.868,0.191)	-0.193 (-1.019,0.632)	-0.331 (-0.853,0.192)	-0.006 (-0.016,0.005)
Work history	At least temporary unemployed	Ref.	Ref.	Ref.	Ref.	Ref.
	Continuously Employed	0.201 (-0.685,1.087)	0.38 (-0.283,1.043)	0.169 (-0.87,1.208)	-0.659 (-1.309,-0.009) *	-0.004 (-0.016,0.009)
Occupational Physical Activity	Low Intensity	Ref.	Ref.	Ref.	Ref.	Ref.
	Intermediate Intensity	-0.361 (-1.82,1.098)	-0.153 (-1.243,0.937)	0.481 (-1.227,2.189)	-0.12 (-1.202,0.963)	-0.006 (-0.027,0.015)
	High Intensity	1.3 (-0.253,2.853)	1.248 (0.088,2.408) *	1.519 (-0.299,3.337)	0.127 (-1.025,1.279)	-0.01 (-0.033,0.012)

Working hours per week	less than 31 hours	Ref.	Ref.	Ref.	Ref.	Ref.
	31-40 hours	1.509 (-0.245,3.262)	1.345 (0.034,2.655) *	-0.558 (-2.613,1.497)	-0.681 (-1.984,0.623)	-0.013 (-0.038,0.011)
	more than 40 hours	2.483 (0.421,4.546) *	2.075 (0.534,3.616) *	1.17 (-1.246,3.587)	-0.393 (-1.926,1.141)	0.003 (-0.027,0.032)
Working shift	Day job	Ref.	Ref.	Ref.	Ref.	Ref.
	Evening/shift	0.385 (-0.819,1.588)	0.243 (-0.662,1.148)	0.494 (-0.92,1.909)	-0.077 (-0.972,0.817)	-0.005 (-0.022,0.012)
Work attitude		0.045 (-0.083,0.174)	0.033 (-0.064,0.129)	0.076 (-0.075,0.228)	0.002 (-0.091,0.095)	0 (-0.002,0.002)
Work engagement		0.01 (-0.037,0.056)	0.01 (-0.025,0.044)	0.04 (-0.015,0.094)	-0.008 (-0.043,0.027)	0 (-0.001,0.001)
Job security	No	Ref.	Ref.	Ref.	Ref.	Ref.
	Yes	1.06 (-0.274,2.393)	0.617 (-0.384,1.617)	0.535 (-1.031,2.1)	-0.668 (-1.653,0.317)	-0.012 (-0.031,0.007)

Fully adjusted models for: BMI, alcohol consumption, educational level, leisure-time physical activity and smoking.
*Relevant confidence intervals.

Supplementary Table 7. Men adjusted linear regression coefficient estimates with 95% confidence interval for the epigenetic age and pace of aging.

		HorvathAA	HannumAA	PhenoAgeAA	GrimAgeAA	DunedinPoAm
		Estimates (95% CI)	Estimates (95% CI)	Estimates (95% CI)	Estimates (95% CI)	Estimates (95% CI)
Job Status	permanent	Ref.	Ref.	Ref.	Ref.	Ref.
	temporary	0.119 (-2.855,3.093)	0.354 (-1.849,2.558)	0.786 (-2.598,4.169)	0.031 (-2.222,2.285)	0.019 (-0.022,0.059)
	unemployed	-0.225 (-2.213,1.763)	-0.11 (-1.584,1.363)	0.276 (-1.986,2.538)	-0.264 (-1.771,1.243)	-0.003 (-0.03,0.024)
Employer	private employer	Ref.	Ref.	Ref.	Ref.	Ref.
	state/municipality	-0.581 (-1.878,0.716)	-0.246 (-1.217,0.724)	-0.232 (-1.732,1.269)	-0.451 (-1.43,0.528)	-0.005 (-0.023,0.013)
Occupational group	Blue collars	Ref.	Ref.	Ref.	Ref.	Ref.
	White collars	0.433 (-0.784,1.65)	0.118 (-0.794,1.03)	-0.283 (-1.705,1.139)	-1.245 (-2.164,-0.325) *	-0.015 (-0.032,0.001)
Job control		0.175 (-0.535,0.885)	0.001 (-0.529,0.531)	-0.316 (-1.14,0.508)	-0.029 (-0.586,0.527)	-0.007 (-0.017,0.003)
Job demand		-0.149 (-0.932,0.633)	-0.106 (-0.69,0.478)	-0.716 (-1.619,0.187)	-0.347 (-0.957,0.264)	-0.012 (-0.023,-0.001) *
Job strain Linear		-0.511 (-1.815,0.794)	-0.168 (-1.142,0.807)	-0.335 (-1.845,1.175)	-0.447 (-1.468,0.574)	-0.006 (-0.025,0.012)
Job strain	Low strain	Ref.	Ref.	Ref.	Ref.	Ref.
	active work	-0.211 (-1.546,1.125)	-0.035 (-1.028,0.958)	-1.458 (-2.993,0.078)	-0.233 (-1.276,0.811)	-0.016 (-0.035,0.002)
	passive work	-0.095 (-1.526,1.336)	0.102 (-0.962,1.167)	-0.458 (-2.103,1.188)	0.625 (-0.493,1.744)	0.012 (-0.008,0.032)
	high strain	0.965 (-0.873,2.803)	1.104 (-0.263,2.47)	0.212 (-1.9,2.325)	0.105 (-1.331,1.541)	0.004 (-0.021,0.03)
Job strain quotient		-0.749 (-2.776,1.278)	-0.365 (-1.878,1.148)	-0.585 (-2.93,1.761)	-0.454 (-2.041,1.133)	-0.005 (-0.034,0.023)
Job strain tertile	Low strain	Ref.	Ref.	Ref.	Ref.	Ref.
	Intermediate strain	-0.813 (-2.053,0.427)	-0.476 (-1.401,0.448)	-0.866 (-2.3,0.569)	0.367 (-0.601,1.335)	0.009 (-0.008,0.027)
	high strain	-0.137 (-1.517,1.243)	0.25 (-0.779,1.279)	-0.011 (-1.608,1.585)	-0.586 (-1.664,0.491)	0 (-0.02,0.02)
Effort		-0.113 (-0.916,0.689)	-0.169 (-0.768,0.43)	0.253 (-0.682,1.188)	0.424 (-0.203,1.05)	0.011 (-0.001,0.022)

Reward		0.389 (-0.586,1.364)	0.462 (-0.264,1.187)	0.323 (-0.805,1.451)	-0.488 (-1.256,0.28)	-0.007 (-0.021,0.007)
Effort-Reward Imbalance		-0.283 (-1.565,0.998)	-0.377 (-1.331,0.578)	0.016 (-1.466,1.499)	0.765 (-0.242,1.771)	0.018 (-0.001,0.036)
Overcommitment		0.85 (-0.032,1.731)	0.575 (-0.086,1.237)	0.721 (-0.306,1.749)	0.3 (-0.394,0.994)	0.008 (-0.005,0.02)
Work history	At least temporary unemployed	Ref.	Ref.	Ref.	Ref.	Ref.
	Continuously Employed	0.258 (-0.803,1.319)	0.08 (-0.707,0.866)	-0.47 (-1.677,0.737)	-0.005 (-0.81,0.799)	-0.006 (-0.02,0.009)
Occupational Physical Activity	Low Intensity	Ref.	Ref.	Ref.	Ref.	Ref.
	Intermediate Intensity	-0.258 (-1.859,1.343)	-0.132 (-1.33,1.066)	0.764 (-1.122,2.65)	1.651 (0.4,2.901) *	0.013 (-0.01,0.036)
	High Intensity	-1.775 (-3.283,-0.267) *	-1.22 (-2.348,-0.092) *	0.089 (-1.688,1.865)	0.378 (-0.8,1.556)	-0.012 (-0.034,0.009)
Working hours per week	less than 31 hours	Ref.	Ref.	Ref.	Ref.	Ref.
	31-40 hours	1.854 (-1.247,4.955)	0.903 (-1.416,3.223)	-0.512 (-4.139,3.115)	-0.497 (-2.931,1.938)	0.015 (-0.029,0.058)
	more than 40 hours	2.095 (-1.089,5.278)	1.072 (-1.309,3.454)	-0.609 (-4.332,3.115)	-0.884 (-3.383,1.616)	0.005 (-0.04,0.05)
Working shift	Day job	Ref.	Ref.	Ref.	Ref.	Ref.
	Evening/shift	0.129 (-1.211,1.468)	0.339 (-0.661,1.339)	0.229 (-1.333,1.792)	0.446 (-0.598,1.491)	0.021 (0.002,0.039) *
Work attitude		-0.014 (-0.172,0.144)	-0.033 (-0.15,0.084)	-0.144 (-0.323,0.034)	0.073 (-0.046,0.192)	-0.001 (-0.003,0.001)
Work engagement		0.012 (-0.04,0.064)	0.005 (-0.034,0.043)	-0.021 (-0.081,0.04)	-0.015 (-0.056,0.025)	-0.001 (-0.001,0)
Job security	No	Ref.	Ref.	Ref.	Ref.	Ref.
	Yes	1.293 (-0.13,2.716)	1.132 (0.072,2.191) *	1.407 (-0.253,3.067)	0.69 (-0.429,1.809)	0.005 (-0.015,0.026)

Fully adjusted models for: BMI, alcohol consumption, educational level, leisure-time physical activity and smoking.
*Relevant confidence intervals.
